# Intra and inter: Alterations in functional brain resting‐state networks after peripheral nerve injury

**DOI:** 10.1002/brb3.1747

**Published:** 2020-07-12

**Authors:** Xiang‐Xin Xing, Xu‐Yun Hua, Mou‐Xiong Zheng, Zhen‐Zhen Ma, Bei‐Bei Huo, Jia‐Jia Wu, Shu‐Jie Ma, Jie Ma, Jian‐Guang Xu

**Affiliations:** ^1^ School of Rehabilitation Science Shanghai University of Traditional Chinese Medicine Shanghai China; ^2^ Department of Traumatology and Orthopedics Yueyang Hospital Shanghai University of Traditional Chinese Medicine Shanghai China; ^3^ Yangzhi Rehabilitation Hospital Tongji University Shanghai China; ^4^ Center of Rehabilitation Medicine Yueyang Hospital Shanghai University of Traditional Chinese Medicine Shanghai China

**Keywords:** functional connectivity, independent component analysis, peripheral nerve injury, resting‐state network

## Abstract

**Introduction:**

Numerous treatments suggest that brain plasticity changes after peripheral nerve injury (PNI), and most studies examining functional magnetic resonance imaging focused on abnormal changes in specific brain regions. However, it is the large‐scale interaction of neuronal networks instead of isolated brain regions contributed to the functional recovery after PNI. In the present study, we examined the intra‐ and internetworks alterations between the related functional resting‐state networks (RSNs) in a sciatic nerve injury rat model.

**Methods:**

Ninety‐six female rats were divided into a control and model group. Unilateral sciatic nerve transection and direct anastomosis were performed in the latter group. We used an independent component analysis (ICA) algorithm to observe the changes in RSNs and assessed functional connectivity between different networks using the functional networks connectivity (FNC) toolbox.

**Results:**

Six RSNs related to PNI were identified, including the basal ganglia network (BGN), sensorimotor network (SMN), salience network (SN), interoceptive network (IN), cerebellar network (CN), and default mode network (DMN). The model group showed significant changes in whole‐brain FC changes within these resting‐state networks (RSNs), but four of these RSNs exhibited a conspicuous decrease. The interalterations performed that significantly decreased FNC existed between the BGN and SMN, BGN and IN, and BGN and DMN (*p* < .05, corrected). A significant increase in FNC existed between DMN and CN and between CN and SN (*p* < .05, corrected).

**Conclusion:**

The results showed the large‐scale functional reorganization at the network level after PNI. This evidence reveals new implications to the pathophysiological mechanisms in brain plasticity of PNI.

## Graphical abstract

Brain plasticity contributes to the sensorimotor recovery after peripheral nerve injury (PNI). It is the large‐scale interaction of neuronal networks instead of isolated brain regions contributed to the functional recovery after peripheral nerve injury. However, little investigations concentrated on the alteration intra and inter the functional brain resting‐state networks. We are the pioneer who tried to use ICA to explore the intrinsic changes and the interactive connectivity between the related functional resting‐state networks in peripheral nerve injury rat models. The result showed that significant intrinsic alteration in identified resting‐state networks, and these networks exhibited a conspicuous decrease in the PNI group. These decline of the activity within brain functional networks may have a direct correlation with functional loss after PNI. And the significant interactivity alterations existed between these resting‐state networks; the alterations verified the damage in motor‐related functional neural circuits and may offer new implications into the neuropathophysiological mechanisms of PNI.

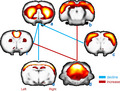

## Significance Statement

Brain plasticity contributes to the sensorimotor recovery after peripheral nerve injury (PNI), Neuroimaging studies often focused on abnormal changes in isolated brain regions but not the functional reorganization of brain networks. The significance of this work is revealed that intra‐ and internetwork alterations between the related functional resting‐state networks (RSNs) in PNI rat models. Our study suggested that activity was declined within brain functional networks in model group, and significant interactivity alterations were existed between these RSNs which verified the damage in motor‐related functional neural circuits. Our result can strengthen theoretical basis for brain plasticity after PNI.

## 1 INTRODUCTION

Peripheral nerve injury is characterized by muscular atrophy, persistent pain, paresthesia, weakness, and paralysis (Michal, Eldad, & Daniel, 2011). More than $150 billion every year is used to treat 20 million American PNI patients (Grinsell & Keating, 2014). A large amount of research has focused on the local changes in the damaged peripheral nerve itself, such as signaling pathways, cellular mechanisms, biomaterial nerve scaffolds, and surgical repairing techniques (Chen, Piao, & Bonaldo, 2015; Davis et al., 2009; Diana et al., 2012; Dominguez, Rivat, Pommier, Mauborgne, & Pohl, 2008; Tseng et al., 2016). Microsurgical techniques and neural regeneration‐promoting approaches remain the common methods for repairing the damaged nerves and restore function of the paralyzed limbs (Kuphal, Fibuch, & Taylor, 2007; Lever, Pheby, & Rice, 2010). Nevertheless, functional recovery after PNI is often incomplete and far from satisfactory (Du, Chen, Qing, Yang, & Jia, 2018).

Recent accumulating evidence suggests that brain plasticity contributes to the sensorimotor recovery after PNI (Alvarado, Szyf, & Millecamps, 2013; Qiu et al., 2013). Mohanty summarized that brain plasticity after nerve injury consisted of two stages (Mohanty, Bhat, & Devi, 2015). The first stage is denervation, in which the cortical area represented by the damaged nerve is invaded by the surrounding area. The second is reinnervation, in which the axonal reorientation at the site of PNI and the defined organizational cortical area exhibit ill‐defined transformations. Hisham et al. used transcranial magnetic stimulation (TMS) to evaluate four brachial plexus injured patients who underwent intercosto‐musculocutaneous nerve transfer (Hisham and Hollis, 2018). TMS showed that the original bicep cortical area regained control of the biceps muscle via the intercostal neurons after a period of time.

Functional magnetic resonance imaging (fMRI) is an important neuroimaging technique that is used for dynamic observations of spontaneous activity in the brain (Biswal, 2012; Logothetis, 2008). However, most neuroimaging studies focused on abnormal changes in one or several specific brain regions. For example, Fornander, Nyman, Hansson, Brismar, & Engström, 2016) found that activity in the ipsilateral primary sensory cortex increased significantly during tactile stimulation in the median nerve of the injured hand of patients Onishi et al. (2018) reported that, significant signal changes occurred in the amygdala, cingulate cortex, basal ganglia, and insular cortex in rats one week after sciatic nerve injury. Our group found that the activation mode of the supplementary motor area played a key role in brain remodeling and clinical functional recovery after PNI (Lu et al., 2016). Electroacupuncture intervention in rats after PNI showed different remodeling patterns in the somatosensory cortex from the model group (Wu, Yechen, Hua, Shujie, & Jianguang, 2018). The amplitudes of low‐frequency fluctuation were significantly increased in the ipsilateral insular of facial synkinesis patients (Wu et al., 2019). The ^18^F‐FDG exhibited significantly increased intake in the contralateral anterodorsal hippocampus and ipsilateral dorsolateral thalamus after right brachial plexus avulsion (Shen et al., 2019). However, the hypothesis of these studies was that isolated brain regions contributed to brain plasticity. Actually, behavior‐related brain activation patterns depended on the integration of brain networks, which consisted of several homogenous brain regions. Similarly, the modifications in the central nerve system after PNI depended on the large‐scale interaction of neuronal networks (Bhat, Indira, Bharti, & Panda, 2017; Feng et al., 2015), such as the DMN, the executive control network (ECN), and the salience network (SN).

Independent component analysis (ICA) is commonly used for identifying latent networks and describing the characteristic spatial patterns and temporal dynamics in most situations (Fox & Raichle, 2007; Wang & Guo, 2019). It decomposes fMRI signals into potential spatial source signals, which correspond to various functional networks. These specific and highly reliably networks were called RSNs. ICA supplies an approach to investigate the connectivity in the whole brain. It has been successfully applied to assess the synchronous fluctuations of intrinsic activity in brain, which is important to communication and collaboration (Feng et al., 2015; Lin, Wu, Liu, Lv, & Yang, 2016).

In this study, we used ICA to examine the intra‐ and internetwork alterations between the related functional RSNs in sciatic nerve injury rat models, which is a typical and complete peripheral nerve injury model (Andersson, Oradd, Sultan, & Novikov, 2018). The results strengthen the theoretical basis for brain plasticity after peripheral nerve injury.

## 2 METHODS AND MATERIALS

### 2.1 Animals

Ninety‐six healthy adult female clean‐grade Sprague Dawley (SD) rats were involved in our study. All rats were aged 6–8 weeks, weighed 180–240 g, and were provided by Shanghai Slack Laboratory Animal Limited Liability Company (Shanghai, China). There is no evidence confirmed that any significant difference between genders in PNI and regeneration, according to a wide review of available literature and our previous studies on peripheral nerve injury in rats, female rats were preferred (Afshari et al., 2018; Vergara, Romano, Stanca, La Pesa, & Maffia, 2018). And the same sex could eliminate possible result differences due to gender. The rats were kept in a laboratory environment with a 12/12 hr light‐dark cycle at 20–22°C and provided with adequate food and water. The rats were kept for 7 days for acclimation to the new situation before any experiments were started.

The Animal Ethics Committee of Shanghai University of Traditional Chinese Medicine approved the study. All procedures and protocols were performed in accordance with the Guide for the Care and Use of Laboratory Animals described by the U.S. National Institutes of Health.

### 2.2 PNI procedure

A total of 96 rats were randomly assigned into two groups: controls (*n* = 24), models (*n* = 72). In the PNI model groups, sciatic nerve transection and direct anastomosis were applied to the right hindlimb in each rat. First, the rats were anesthetized with an intraperitoneal injection of sodium pentobarbital (40 mg/kg) and placed on a clean operating table in a prone position. The hair was shaved under the sciatic tubercle of the right hip 5 mm, and an incision was made along the route of sciatic nerve. Under a 10‐fold microscope, the right sciatic nerve trunk was exposed and separated from the gluteal muscle. It was transected with a blade 1 cm below the lower edge of the piriformis muscle. The sciatic nerve was repaired via epineurium suture with 11‐0 single‐strand nylon wires. And the control group without treatment.

### 2.3 MRI image acquisition

Functional magnetic resonance imaging scans of the brain were performed 4 weeks after surgery using a Bruker 7T magnetic resonance system (Bruker Corporation) with a coil for two groups. After 2.5% isoflurane‐induced anesthesia, rats were fixed on the scanner and given a continuous 1.5%–2% isoflurane anesthesia with ventilator support and respiratory monitoring. The following interlayer scanning echo‐plane imaging parameters were used: flip angle = 90°, slice thickness = 0.3 mm, repetition time = 3,000 ms, and there are 200 time points in image acquisition. The duration = 200 × 3 s; in other words, the duration is 10 min. Echo time = 20 ms, number of averages = 1, field of vision (FOV) = 32 × 32 mm^2^, and matrix = 64 × 64 voxels.

### 2.4 fMRI data preprocessing

Data preprocessing procedures were performed using the Statistical Parametric Mapping 12 (SPM 12) toolbox (http://www.fil.ion.ucl.ac.uk/spm/) based on the MATLAB 2014a platform. At first, we removed the first five time points from the data preprocessing and expanded the images by 10 × 10 × 10 times to match the size of the human brain, which made it possible to develop processing algorithms originally designed for human data. The amplified procedure only changed the dimension descriptor fields in the file header without interpolation. Second, the none‐brain tissue was stripped manually before further preprocessing. FMRI images were corrected from the temporal bias of slice acquisition using the slice timing procedure. The images were spatially realigned with rigid‐body transformations to correct the misplacement of voxels, which was caused by in‐scanner head motion. The standard brain template in Schwarz’ study was adopted to achieve normalization of the standard space, and the voxel size for normalized images was 2.06 × 2.06 × 2 mm (Schwarz et al., 2006). And following, the images were smoothed by a full width at half maximum quadruple as the voxel size (8.24 × 8.24 × 8 mm). The further preprocessing includes temporal bandpass filtering (0.01–0.1 Hz) to decrease the low‐frequency drift.

### 2.5 The intranetwork alteration of RSNs

The preprocessed data of the two groups were combined into one group. Group spatial ICA was performed to analyze the combined data using the GIFT software (http://trendscenter.org/software/gift/) (Calhoun, Adali, Pearlson, & Pekar, 2001). The procedures included three steps: dimension reduction by principle component analysis (PCA), ICA decomposition, and back reconstruction for individual level components (Bell & Sejnowski, 1995). A two‐level PCA was performed to reduce the dimensionality of the data. The information‐maximization (infomax) algorithm was used as an independent component estimation. The IC number was identified to be 20 according to the minimum description length criteria and previous studies (Calhoun et al., 2001; Hutchison, Mirsattari, Jones, Gati, & Leung, 2010). Then, the data were decomposed into 20 components using the infomax algorithm. This analysis was repeated 100 times to achieve robust and accurate results. Next, the ICs at the group level (both spatial maps and time courses) were back reconstructed for each subject, and the ICA‐determined networks were converted to Z‐maps before entering group statistics to obtain voxel values comparable across subjects. Six meaningful RSNs were identified as anatomically and functionally classical RSNs via visual inspection (Bajic, Craig, Mongerson, Borsook, & Becerra, 2017). The individual subject spatial maps for each selected RSN were converted to *Z*‐values (Song et al., 2011). For each selected RSN, a voxel‐wise one‐sample *t* test was employed to determine the group spatial map for all subjects (*p* < .05, FDR corrected, false discovery rate), and the statistically thresholded *t*‐value map was used to define brain regions that belonged to the RSN. Differences between the control and model groups were then examined using a voxel‐wise two‐sample *t* test.

### 2.6 The internetworks analysis of RSNs (FNC analysis)

According to ICA algorithm, the time courses of cortical areas within one IC are synchronous, and the time courses of each RSN were extracted and used to calculate the temporal correction. Although the components were spatially independent, significant temporal correlations could exist between them. As an extension of the ICA, the functional network connectivity (FNC) toolbox (http://mialab.mrn.org/software/#fnc) was employed to examine the temporal relationships between brain networks. Corresponding to the significant correlation combinations, the average time lags, which represent the amount of delay between the time courses of the two correlated RSNs, were calculated for each group. The maximum time lag was set to 6 s. One‐sample *t* test (*p* < .05, FDR corrected) for each group and two‐sample *t* test (*p* < .05, FDR corrected) for group comparisons were performed on all possible combinations.

## 3 RESULT

All the rats survived after sciatic nerve transection and repair surgery. Reddening and swelling around the wound was only observed in three rats within one post‐PNI week. And the wound completely recovered in all rats without obvious infection. There was no abnormal image after data acquisition and preprocessing.

### 3.1 The functional network maps

**Figure 1:**
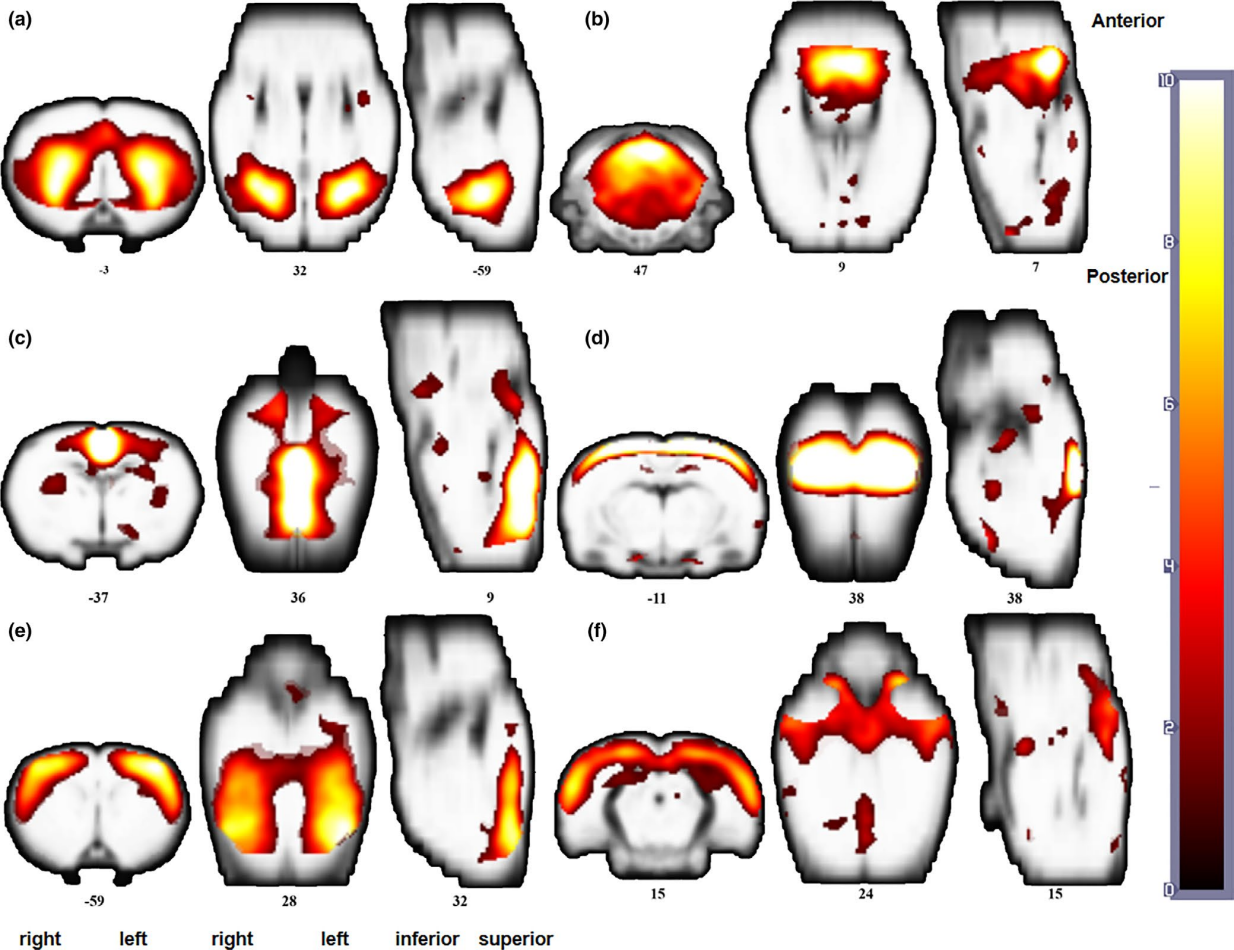
Sagittal, coronal, and axial views of spatial maps for each RSN. (a) Basal ganglia network (BGN), (b) cerebellar network (CN), (c) default mode network (DMN), (d) interoceptive network (IN), (e) sensorimotor network (SMN), (f) salience network (SN). Each RSN map was obtained using a one‐sample *t* test across all individual IC patterns (*p* < .05, FDR corrected). The right side of the image corresponds to the right brain hemisphere. RSN, resting‐state networks

Six RSNs were determined using the ICA algorithm, including the basal ganglia network (BGN), sensorimotor network (SMN), SN, interoceptive network (IN), cerebellar network (CN), and default mode network (DMN) (Figure 1). These functional networks were identified according to previous studies using ICA analysis (Bajic et al., 2017; Becerra, Pendse, Chang, Bishop, & Borsook, 2011; Sierakowiak et al., 2015). Further analyses were performed based on these RSNs.

### 3.2 The intranetwork alterations in identified RSNs in PNI rats

**Figure 2:**
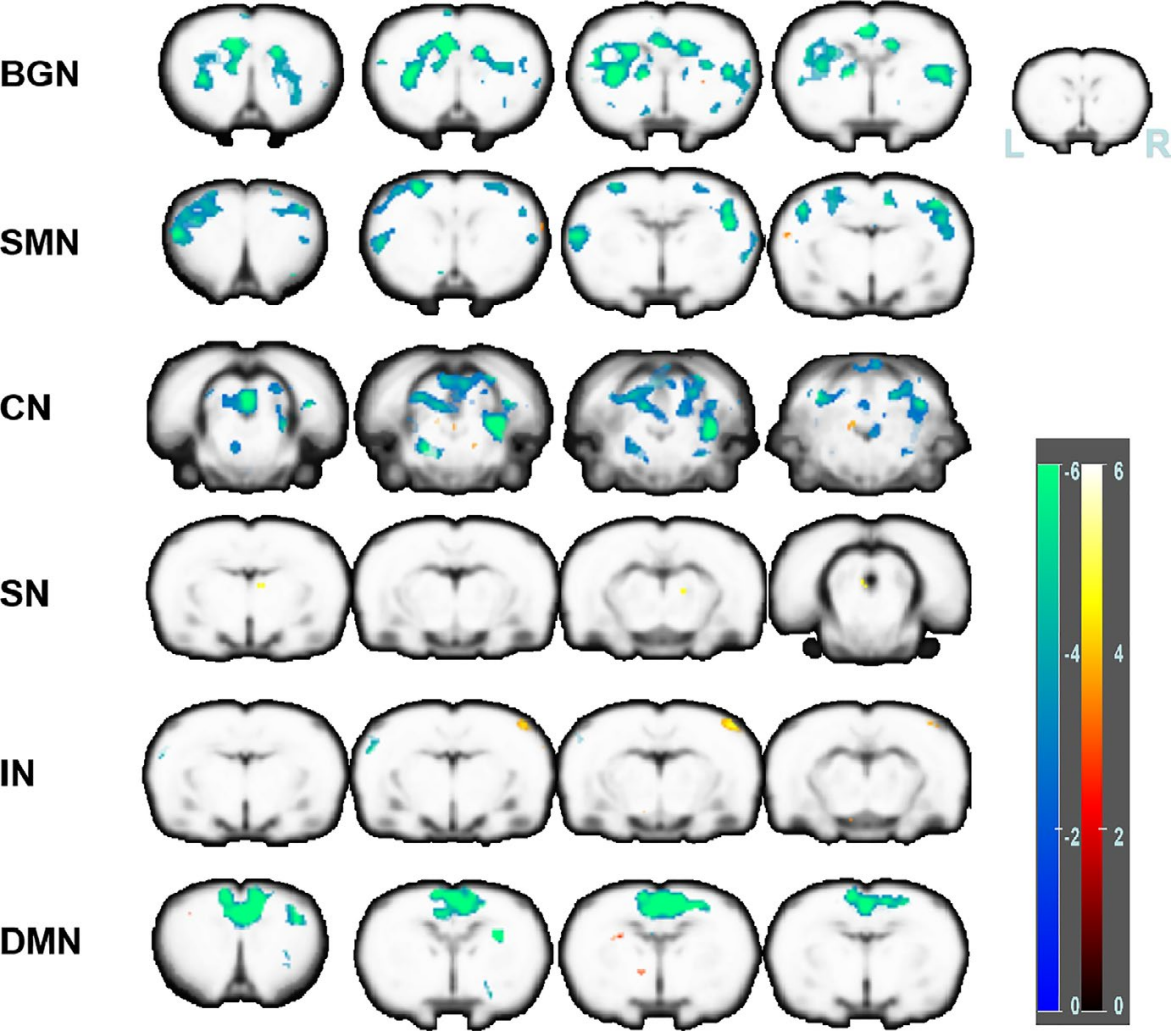
Results of RS FC analysis between control and model group. Altered FC in the basal ganglia network, sensorimotor network, cerebellar network, salience network, interoceptive network and default mode network. The hot color denotes higher functional activity in the model group compared with the control group, and the winter color denotes lower functional activity in the model group

The results of two‐sample *t* tests between the PNI and control groups are shown in Figure 2 and Table 1. The significance level of the *t*‐value in BGN, CN, IN, DMN, and SN were set at *p* < .05 (FDR, corrected). And the SMN did not pass FDR correction We found significant changes in wholebrain FC changes in these RSNs, but four of these networks exhibited a conspicuous decrease in the model group. In the BGN, activities of bilateral caudate, putamen, and corpus collosum and the left cingulate cortex were significantly decreased. In the DMN, the activities of right cingulate cortex, motor cortex, caudate, and putamen were decreased. In the SN, the activity of the right insular cortex was significantly decreased, and the bilateral cingulate cortex was increased. In the IN, the activities of the left somatosensory cortex were significantly decreased, but the right somatosensory cortex was significantly increased.

**Table 1:** Differences between model group and controls in resting state functional connectivity of all networks with potential functional relevance

RSNs	Region label	Voxel size	*t*‐Value	MNI coordinates		
				*x*	*y*	*z*
BGN	L_Cortex_Cingulate	204	−5.227	−9	13	−57
	L_Caudate_Putamen	200	−4.651	−38	−7	−49
	R_Caudate_Putamen	117	−4.2391	18	13	−45
	L_Cortex_Somatosensory	19	−3.7601	−52	3	−49
	L_Corpus_Collosum	17	−3.755	−30	−5	−71
	R_Cortex_Somatosensory	141	4.3814	26	32	−21
CN	L_Cortex_Retrosplenial	24	−4.9152	−5	28	−11
	R_Periaqueductal_Grey	38	−4.4918	1	1	27
	R_Hippocampus_Postero_Dorsal	37	−4.491	47	1	17
	L_Caudate_Putamen	13	−4.4103	−26	9	−53
	L_Superior_Colliculus	14	−4.0734	−5	9	19
	R_Cortex_Somatosensory	20	4.7166	36	21	−31
	R_Mesencephalic_Region	28	4.213	18	−32	9
SN	L_Cortex_Orbitofrontal	16	−5.1018	−40	−17	−69
	R_Corpus_Collosum	11	−4.8155	59	1	−1
	R_Cortex_Medial_Prefrontal	11	5.2964	7	−7	−77
	R_Thalamus_Midline_Dorsal	12	4.928	9	−5	−23
IN	L_Cortex_Somatosensory	62	−5.6077	−61	11	−17
	R_Cortex_Somatosensory	114	4.8418	57	30	−11
DMN	R_Cortex_Cingulate	1752	−5.3712	5	20	−65
	R_Cortex_Motor	1752	−5.3009	12	30	−37
	R_Caudate_Putamen	88	−5.0817	38	−1	−55
	R_Cortex_Retrosplenial	1752	−4.9036	1	34	−11
	L_Cortex_Visual	33	−3.1862	−21	30	25
	R_Cortex_Somatosensory	10	−2.6967	44	17	−67
	L_Corpus_Collosum	15	4.0541	−59	3	15
	R_Hippocampus_Postero_Dorsal	16	3.4296	12	19	−1
	L_Hippocampus_Postero_Dorsal	32	3.3747	−48	9	9
	L_Caudate_Putamen	13	3.0699	−34	5	−37

Abbreviations: BGN, basal ganglia network; CN, cerebellar network; DMN, default mode network; FNC, functional networks connectivity; IN, interoceptive network; RSN, resting‐state networks; SMN, sensorimotor network; SN, salience network.

### 3.3 The interactive alterations between the RSNs

**Figure 3:**
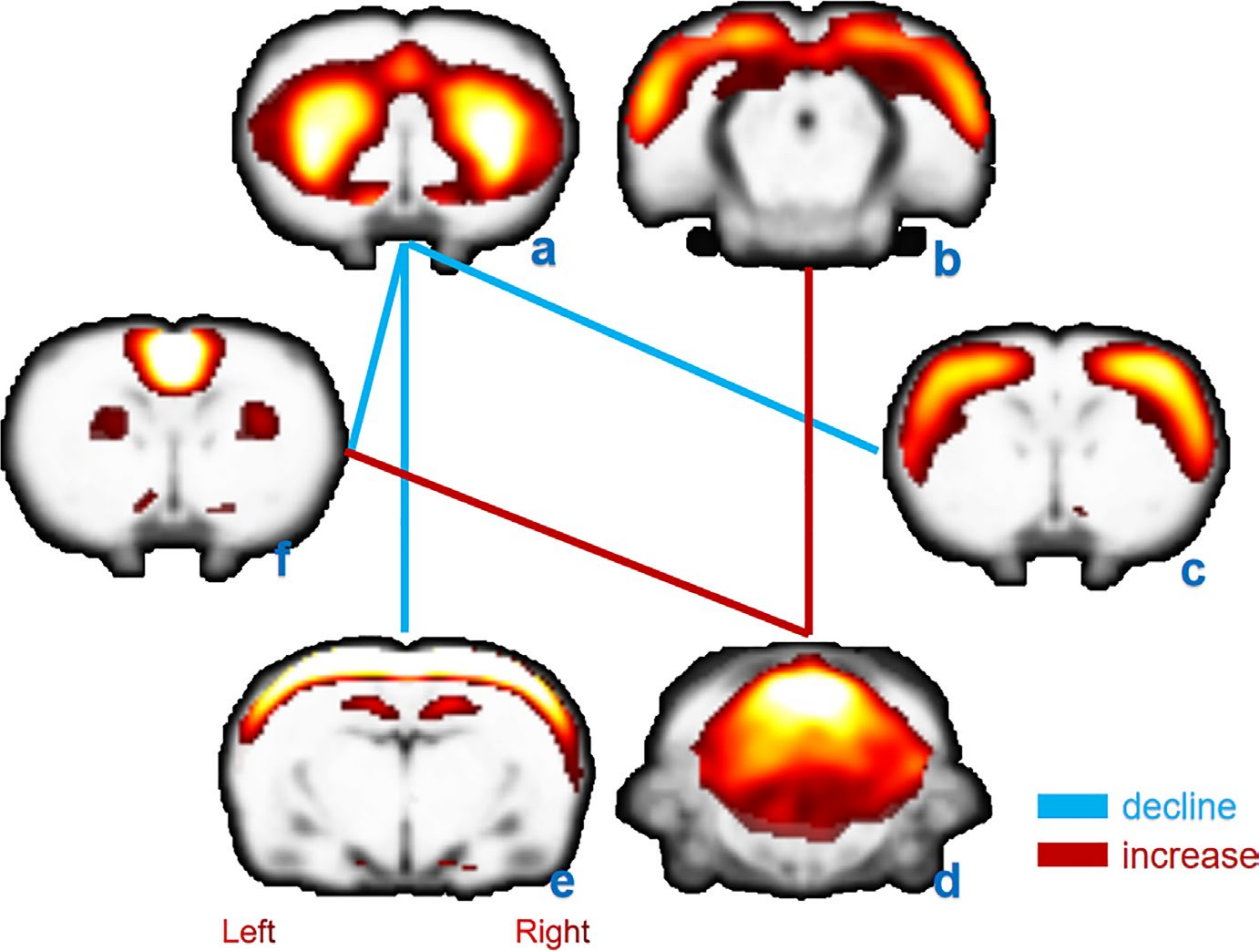
PNI‐related differences in brain functional network connectivity between RSNs. (a) Basal ganglia network, (b) salience network, (c) Sensorimotor Network, (d) cerebellar network, (e) interoceptive network, (f) default mode network. Relative decreased connectivity strength in the model group is displayed by the blue line, and increases are displayed by the red line. PNI, peripheral nerve injury; RSN, resting‐state networks

**Figure 4:**
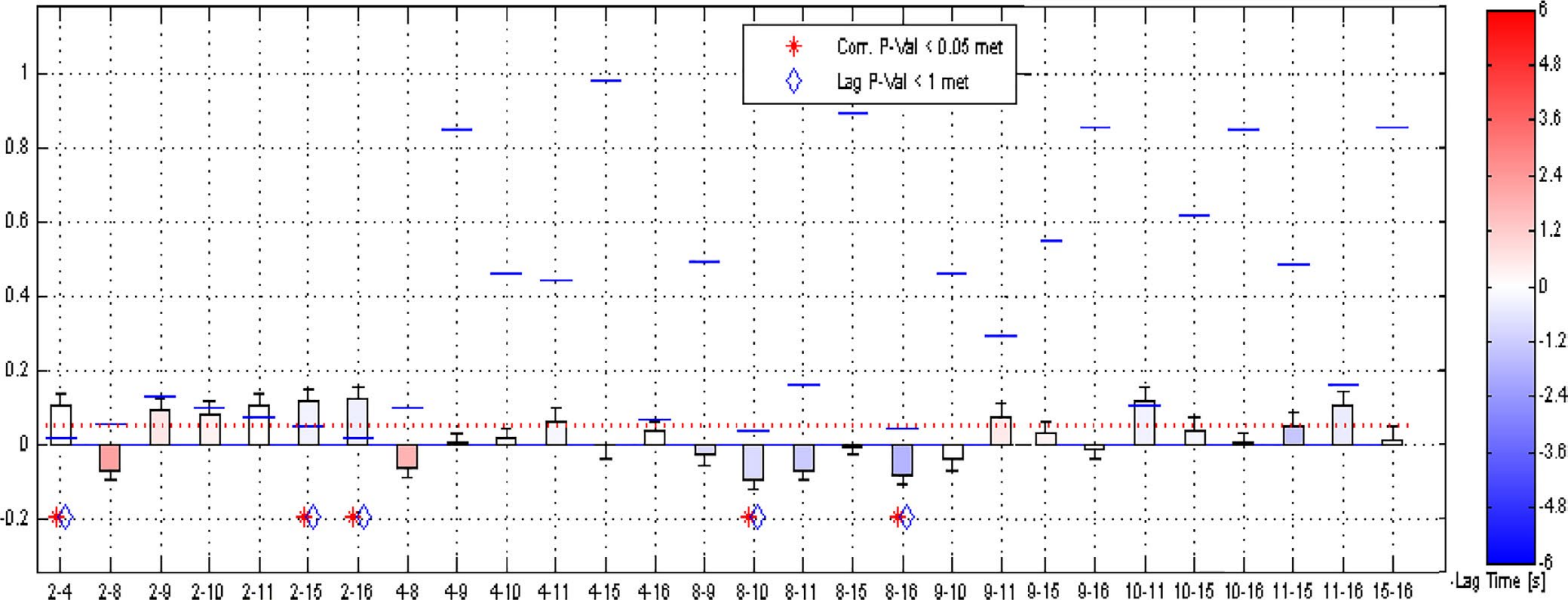
Results of component combination among RSNs. The blue lines show the correlation of *p*‐value; the red dotted line shows the user *p*‐value threshold. 2: BGN; 4: SMN; 8: CN; 10: SN; 15: IN; 16: DMN; and other numbers are not identified resting‐state networks. BGN, basal ganglia network; CN, cerebellar network; DMN, default mode network; IN, interoceptive network; RSN, resting‐state networks; SMN, sensorimotor network; SN, salience network

The results of FNC analyses between RSNs are shown in Figure 3. The significant level of difference between two RSNs correlations was set at *p* < .05 (corrected) in Table. 2 and Figure 4. Significantly decreased connectivity was found between SMN and BGN, BGN and IN, BGN and DMN, and increased connectivity was found between DMN and CN and between CN and SN.

**Table 2:** Significant correlations found among the identified RSNs

FNC		Control	Model	Control vs. Model
RSN1	RSN2	*p*‐Value^a^	*p*‐Value^a^	*p*‐Value^b^
BGN	SMN	<.05	<.05	<.05^c^
	CN	<.05	<.05	NS
	SN	<.05	<.05	NS
	IN	<.05	<.05	<.05^c^
	DMN	<.05	<.05	<.05^c^
SMN	CN	<.05	<.05	NS
	SN	<.05	<.05	NS
	IN	<.05	<.05	NS
	DMN	<.05	<.05	NS
CN	SN	<.05	<.05	<.05^d^
	IN	<.05	<.05	NS
	DMN	<.05	<.05	<.05^d^
SN	IN	<.05	<.05	NS
	DMN	<.05	<.05	NS
IN	DMN	<.05	<.05	NS

Abbreviations: BGN, basal ganglia network; CN, cerebellar network; DMN, default mode network; FNC, functional networks connectivity; IN, interoceptive network; NS, not significant; RSN, resting‐state networks; SMN, sensorimotor network; SN, salience network.

^a^ One‐sample *t* test;

^b^ Two‐sample *t* test (Significant between‐group connectivity differences. Significant *p*‐values are surviving false rate correction for multiple comparison);

^c^ Model > control;

^d^ Control > model.

## 4 DISCUSSION

The present study investigated the intranetwork and interactive alterations of brain networks in PNI rats based on rs‐fMRI data using ICA and FNC algorithms. Our study confirmed decreased activity in the PNI group within RSNs and the functional connectivity changed between RSNs. These alterations revealed that cortical remodeling was extensive within and across related RSNs after PNI. Although most previous studies focused on the plastic changes in isolated brain regions, recent investigations examined the relationship between behavioral recovery and the homogenous brain RSNs, such as SMN (Sammons & Keck, 2015; Taylor, Anastakis, & Davis, 2009). However, these literatures primarily used FNC analysis to investigate cognitive dysfunction, such as dementia, attention deficit hyperactivity disorder, and schizophrenia (Fu et al., 2019; de Lacy & Calhoun, 2019; White, Joseph, Francis, & Liddle, 2010). Few studies focused on whole‐brain analyses of static FNC after PNI.

Each RSN has a complex internal anatomical structure with special function. The SMN is composed of bilateral sensory, motor, and visual cortexes. The function of the SMN includes the integration of motor, sensory, emotional, and executive control. The IN is similar to the SMN with sensory cortices, and the two networks were classified as sensory and interoceptive networks, respectively. The IN handles information of physiological conditions in the body (Lino, Gautam, Pei‐Ching, James, & David, 2011). The basal ganglia consist of the striatum, globus pallidus, substantia nigra, and subthalamic nucleus (Plenz & Kital, 1999). The striatum receives input from the sensorimotor cortex and cerebellum, and the pallidum sends inhibitory output to motor‐related areas (Bernhard, 1997). Therefore, the BGN is inextricably linked to spontaneous movement. The CN is primarily composed of the cerebellum and other brainstem areas, such as the periaqueductal gray and raphe nuclei. The CN plays a critical role in sensory‐motor integration, arousal, and protective processing (Berridge, 2008; Habas et al., 2009). The DMN is composed of prefrontal cortical regions, the cingulate cortex, and retrosplenial cortex (Sierakowiak et al., 2015). The SN contains the insular and cingulate cortices (Valerie et al., 2012). The current study revealed reduced intranetwork activities in the model group in the SMN, BGN, IN, DMN, and CN. These results are consistent with dysfunction of motor and sensory after peripheral nerve injury (Michal et al., 2011). These results are also similar to the results of blood oxygen‐dependent level signals in human and animal models in previous studies (Onishi et al., 2018; Wu et al., 2018). Extensive decline of the activity within brain functional networks may have a direct correlation with functional loss after PNI.

Notably, the BGN played a special role in our experiment. The FNC results showed that the connectivity between BGN and SMN, BGN and IN, BGN, and DMN decreased significantly in the model group. It is generally accepted that there is an existing neural loop between the thalamo‐cortex‐basal ganglia that contributes to the motor execution of sensory integration and sensory‐motor feedback (Cole, Sudhir, & Walter, 2010), which has great significance in the generation of autonomous motion (Filip, Lungu, & Bareš, 2013; Tanji, 1996; Tripathi, Prensa, & Mengual, 2013). The brain regions in BGN and related RSNs also form different neural circuits, such as memory, sensory, and emotion. The decreased connectivity between BGN and the other three RSNs (SMN, IN, and DMN) suggests a disruption of information communication in the neural loop. This condition may be the reason for poor clinical outcome after PNI. The function of the cerebellum consists of motor function support and motor‐related cognitive processes (Bohne, Schwarz, Herlitze, & Mark, 2019). Habas et al. found that the cerebellum contributed to executive control, salience detection, memory, and self‐reflection. Particularly, it was also an important part of the ECN (Habas et al., 2009). These results may explain why the FC between CN and DMN, CN and SN were increased. Coincidentally, the FC between DMN and ECN was also significantly increased in patients with brachial plexus avulsion (Feng et al., 2015). These results may suggest that the cooperation between the cerebellum in ECN and DMN is more intimate during cognitive processes after PNI.

We would set up the intervention group in subsequent experiments to make the study more convincing. The 7T magnetic resonance system was used to scan the brain because we needed a higher scan resolution to further study functional connectivity in PNI, such as the 11.2T system. Few researchers focused on ICA and FNC in rodents, and the standards of RSNs are not perfect. Therefore, we could not identify components with a specific function. And in further research, we would reveal the dynamic changes in the brain networks of patients after peripheral nerve injury.

The current study clarified the large‐scale functional reorganization at the network level, and whole brain activities were significantly decreased after PNI. Alterations in connectivity between RSNs verified the damage in motor‐related functional neural circuits. This evidence strengthens the theoretical basis for brain plasticity after peripheral nerve injury.

## CONFLICT OF INTEREST

The authors declare no conflict of interest. None of the authors have a commercial interest in the material presented in this work.

## AUTHOR CONTRIBUTIONS

X.X., and X.H. involved in conceptualization; X.H., M.Z., and S.M. involved in methodology; J.W. contributed to software; Z.M., B.H., and J.M., contributed to validation; X,X., X.H., and M.Z. involved in formal Analysis; X,X., Z.M., B.H., and J.M. involved in investigation; X.X., and X.H. contributed to writing—original draft; M.Z., and J.X. contributed to writing—review & editing; All authors had full access to all the data in the study and take responsibility for the integrity of the data and the accuracy of the data analysis.

## Data Availability

We will share the data at the end of our project. Identified data from this study will be shared upon reasonable request from a qualified investigator.
